# Functional conservation of the grapevine candidate gene *INNER NO OUTER* for ovule development and seed formation

**DOI:** 10.1038/s41438-021-00467-5

**Published:** 2021-02-01

**Authors:** Valentina di Rienzo, Zahra Imanifard, Isabella Mascio, Charles S. Gasser, Debra J. Skinner, Ciro Leonardo Pierri, Martina Marini, Valentina Fanelli, Wilma Sabetta, Cinzia Montemurro, Diana Bellin

**Affiliations:** 1 Department of Soil, Plant and Food Sciences, Section of Genetics and Breeding, University of Bari Aldo Moro, via Amendola 165/A, 70125 Bari, Italy; 2 Spin off Sinagri s.r.l., University of Bari Aldo Moro, via Amendola 165/A, 70125 Bari, Italy; 3 Department of Biotechnology, University of Verona, Strada le Grazie 15, 37134 Verona, Italy; 4 Department of Molecular and Cellular Biology, University of California, Davis, 1 Shields Ave., Davis, CA 95616 USA; 5 Department of Biosciences, Biotechnologies and Biopharmaceutics, Laboratory of Biochemistry Molecular and Structural Biology, University of Bari Aldo Moro, Via E. Orabona 4, 70126 Bari, Italy; 6 Spin off BROWSer S.r.l. (https://browser-bioinf.com/) c/o Department of Biosciences, Biotechnologies, Biopharmaceutics, University of Bari Aldo Moro, Via E. Orabona 4, 70126 Bari, Italy; 7 Institute of Biosciences and Bioresources of the National Research Council (IBBR-CNR), Via Amendola 165/A, 70125 Bari, Italy; 8 Institute for Sustainable Plant Protection–Support Unit Bari, National Research Council of Italy (CNR), Via Amendola 165/A, 70125 Bari, Italy

**Keywords:** Plant breeding, Seed development

## Abstract

Seedlessness represents a highly appreciated trait in table grapes. Based on an interesting case of seedless fruit production described in the crop species *Annona squamosa*, we focused on the *Vitis vinifera INNER NO OUTER (INO)* gene as a candidate. This gene encodes a transcription factor belonging to the *YABBY* family involved in the determination of abaxial identity in several organs. In *Arabidopsis thaliana*, this gene was shown to be essential for the formation and asymmetric growth of the ovule outer integument and its mutation leads to a phenotypic defect of ovules and failure in seed formation. In this study, we identified in silico the *V. vinifera* orthologue and investigated its phylogenetic relationship to *INO* genes from other species and its expression in different organs in seeded and seedless varieties. Applying cross-species complementation, we have tested its functionality in the Arabidopsis *ino*-1 mutant. We show that the *V. vinifera* INO successfully rescues the ovule outer integument growth and seeds set and also partially complements the outer integument asymmetric growth in the Arabidopsis mutant, differently from orthologues from other species. These data demonstrate that VviINO retains similar activity and protein targets in grapevine as in Arabidopsis. Potential implications for grapevine breeding are discussed.

## Introduction

Grapevine (*Vitis vinifera* L.) is one of the most cultivated and appreciated fruit crop trees in many regions of the world. It is cultivated mainly for wine-making, but also for fresh consumption and raisins. Breeding programs are focused either on pathogen resistance or on qualitative traits appreciated by consumers, among which seedlessness for table grape^1^. Two different types of seedless fruits have been observed in grape, caused either by parthenocarpy or stenospermocarpy^1,2^. In parthenocarpy, fruit development occurs in the absence of ovule fertilization, leading to a complete lack of seeds^3^. In stenospermocarpy, ovule fertilization takes place but seed development fails because of the embryo and/or endosperm degeneration. Stenospermocarpic cultivars are not strictly seedless but they contain seminal rudiments or seed traces of different sizes^2^. Genetic studies have mapped grape QTLs for seeds related trait to several genomic regions (see refs. ^4,5^ for a summary). The Sultanine (or Thomson seedless) cultivar contributes to the *SEED DEVELOPMENT INHIBITOR* (*SDI*) locus, the major source of seedlessness exploited for breeding purposes and in commercial grapevine cultivars^6–10^. A recent study has elucidated the molecular basis of this trait demonstrating this is associated with a missense mutation in the MADS-Box gene *VviAGL11* controlling seed coat development and lignification^11^. Besides this major gene, genetic studies indicate that alternative less exploited genes contribute to the seedless phenotype in grapevine.

An interesting case of seedless fruit production was described in the spontaneous mutant *Thai seedless* (*Ts*) of the crop species *Annona squamosa* (sugar apple), belonging to an early divergent angiosperm clade^12^. This mutant produces fully seedless normal size fruits following pollination. The authors demonstrated that failure in seed formation is due to a defect in the ovules which lack the outer of the two normal integuments, phenocopying the Arabidopsis *inner no outer (ino-1)* mutant^13^. They isolated the Annona *INO* orthologue gene and showed that the mutant was indeed associated with the deletion of the *INO* locus, revealing the molecular basis of seedlessness in the *Ts* mutant and providing an interesting candidate with the potential of introducing seedlessness in further crop species.

The *INO* gene encodes a putative transcription factor belonging to the *YABBY* gene family, involved in the determination of abaxial identity in a variety of plant organs^14–16^. *INO* and its role in ovule development were mainly characterized in the model plant species Arabidopsis. Phenotypic analyses in the *ino-1* mutant have shown, besides the lack of the outer of the two integuments which normally cover the nucellus in plant ovules, also the absence of the typical hoodlike structure (amphitropous) characteristic of wild-type ovules. Indeed, the amphitropous configuration is due to the asymmetric growth of the outer integument, and it is therefore lost in the mutant plants that show the micropyle not adjacent, but in a line, with the funiculus. Moreover, *ino-1* plants exhibit a strongly reduced number of viable seeds and, differently from the *Ts* Annona crop mutant, reduced siliques expansion^17–19^. Further molecular characterizations have shown that the *ino-1* mutant phenotype in Arabidopsis was due to a G-to-A transition close to a splice acceptor site of the gene, which leads to a frameshift mutation affecting the coded protein. Investigation on the spatial distribution of *INO* transcript accumulation showed that *INO*, prior to the visible emergence of the integuments, is expressed specifically in the epidermal cells on the abaxial half of ovule primordium, the region corresponding to the site of outer integument initiation. Therefore, INO was suggested to be involved in the polar determination and to work as a primary determinant of abaxial identity in the ovule part from which the outer integument originates through strict control of its expression pattern^13^. Further studies showed that *INO* expression is regulated by a positive autoregulatory loop and that this loop is attenuated by the *SUPERMAN(SUP)* repressor in the adaxial ovule side. Indeed, in the *sup* mutant, the outer integument grows also on the adaxial side of the ovule primordium, resulting in ovules with a nearly radially symmetrical tubular shape^17^. This molecular mechanism directly controls the polar development of ovule outer integument^13,20^.

Beside studies in the model plant Arabidopsis, *INO* orthologues were characterized in a number of different taxa and species. The highly specific expression pattern described in Arabidopsis was compared to the expression pattern in early-diverging bitegmic angiosperms such as *Nymphaea alba* and *Cabomba caroliniana*, from the Nymphaeales order, or *Amborella trichopoda*, from the Amborellales^21–24^. These studies showed that INO expression pattern in these early-diverging lineages exactly parallels that observed in Arabidopsis indicating that exclusive expression of *INO* in the abaxial epidermis of the outer integument is primitive and has been conserved from early stages of angiosperm evolution. Further comparison of *INO* orthologues expression pattern in unitegumic species confirmed the specific expression in the abaxial outermost cell layer also of the single ovule integument, indicating widely conserved INO function across all angiosperms^12,25–27^.

Despite the described conservation in the expression pattern, studies addressing INO functional conservation, including comparative characterization of protein targets and activities are still few. While virus-induced knockdown of expression of the *INO* orthologue in *Nicotiana benthamiana*, a representative of the unitegumic *Solanales* in the asterids clade, inhibited growth of the outermost cell layer of the unique ovule integument, leading to a decrease in both integument extension and ovule curvature, the tomato *SlINO* CDS failed to complement the Arabidopsis *ino-1* mutant phenotype, indicative of divergences in protein targets and activity^27^. Therefore, despite the claim of a widely conserved role for *INO* also to unitegumic species and to near the base of the angiosperms, cross-species complementation did not support that so far.

In this study, starting from the recent characterization of the YABBY gene family in grapevine^28^ we have identified the grapevine *VviINO* gene and investigated its phylogenetic relationship to *INO* orthologues from other species and its expression in different plant organs and seeded and seedless cultivars. Moreover, we conclusively demonstrated that the grapevine orthologue *VviINO* retains similar protein targets and activity in the grape as in Arabidopsis, since it could fully restore the outer integument growth in Arabidopsis *ino-1* mutant. Some specificities in the asymmetric growth were also observed and discussed. These data provide relevant information on a new candidate with potential implications for table grape breeding.

## Results

### *In silico* identification and annotation of the grapevine *VviINO* gene

The genomic organization of the *YABBY* gene family members in *V. vinifera*, including the gene encoding VviINO, was recently described by Zhang et al.^28^. To validate and refine the *VviINO* annotation, structural information for the whole gene family was updated to the newest genome assembly 12X.v2 (Supplementary Table S1). Minor differences in genomic positions, length of genes, related CDS structures and predicted protein sequences were noted according to the different available annotations. However, careful inspection of multi-alignments showed that predicted proteins, according to two latest and most used annotations V2 or VCost.v3^29,30^ were congruent for all family members, with the only exception of VIT_201s0011g00140 which is different in the N-terminal protein portion and VIT_206s0009g00880 missing 2aa in the VCost.v3 protein version compared to V2 annotation (Supplementary Fig. S1). In silico analysis of predicted protein sequences confirmed the presence of nuclear localization signals, in line with the expected function, as well as the presence of the conserved YABBY domain located from 2nd to 162th aa, including both hallmarks motifs of the family, i.e., the Zinc finger toward N-terminal and the helix–loop–helix YABBY domain toward C-terminal protein portion^14–16^ (Supplementary Table S2).

**Fig. 1:**
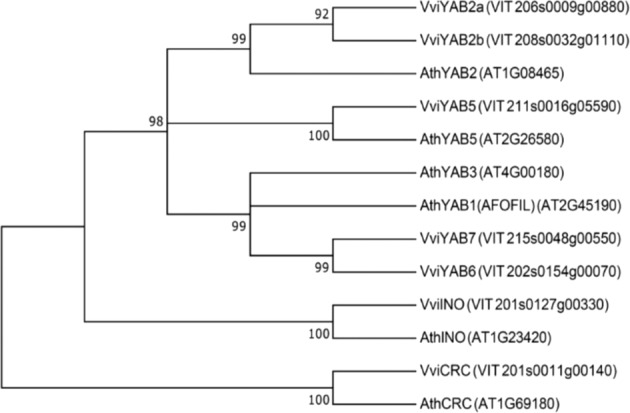
Phylogenetic tree of YABBY family members from *A. thaliana* and *V. vinifera*. Phylogenetic tree was constructed using CDS of *A. thaliana* (*Ath*) and *V. vinifera* (*Vvi*) YABBYs by using the UPGMA method and p-distance to establish orthologous relationships. All positions containing gaps and missing data were eliminated. Low bootstrap support (<70%) is not shown

Direct orthologous relationships between *V. vinifera* and Arabidopsis *YABBY*s were established based on CDS analysis. Among all grape *YABBYs* coding sequences, *VIT_201s0127g00330* located on chromosome 1 formed a clade with *A. thaliana AT1G23420* encoding for *INO*, suggesting this gene as the corresponding grapevine *INO* orthologue (Fig. 1). Distance matrix estimated from both DNA coding sequence and protein sequence alignments confirmed this closer relationship (Supplementary Table S3), consistent with previous analysis and the genomic location in syntenic blocks between grape and Arabidopsis genomes^28^. Following guidelines established by the Super-Nomenclature Committee for Grape Gene Annotation (sNCGGa)^31^, we have revisited the whole grapevine *YABBY* family nomenclature, and propose to rename *VIT_211s0016g05590* as *VviYAB5* according to its highest sequence similarity to Arabidopsis *YAB5*. *VIT_206s0009g00880* and *VIT_208s0032g01110*, showing both highest sequence similarity to Arabidopsis *YAB2*, were renamed as *VviYAB2a* and *VviYAB2b* as a paralogous set of genes located on different chromosomes. Another putative paralogous set, *VIT_202s0154g00070*, and *VIT_215s0048g00550*, showed equal sequence similarity to the Arabidopsis *YAB1* and *YAB3* genes which are recognized as belonging to a subclade in the family^22^. Since no one2one orthologous relationship could be established, we renamed these as *VviYAB6* and *VviYAB7* respectively, using numbers higher than the highest already used for both Vitis and Arabidopsis according to rules defined by sNCGGa^31^. *VIT_201s0011g00140* was finally renamed as *VviCRC* due to the high similarity with the characterized Arabidopsis *CRC*.

### Phylogenetic relationship among INO proteins from different species

Evolutionary relationships for grape INO with species in which this gene was previously characterized were enquired.

INO protein function has been first deeply characterized in the model plant *A. thaliana*, where its role in integument development during ovule formation was widely studied^24^. Sequence comparison revealed that grape INO shares 79% similarity and 69% identity with the Arabidopsis INO. However, while the Arabidopsis *INO* encodes a 231 aa protein^13^ *VviINO* encodes a shorter protein (176 aa). Sequence alignment revealed nearly complete conservation inside both the zinc finger domains as well as in the HLH domain, each presenting only five unconserved amino acids. High conservation was found also in border regions of both conserved domains and in the protein portion among the two domains, while terminal protein portions were more divergent, with a long C-terminal unshared sequence only present in the Arabidopsis INO (Supplementary Fig. S2).

**Fig. 2:**
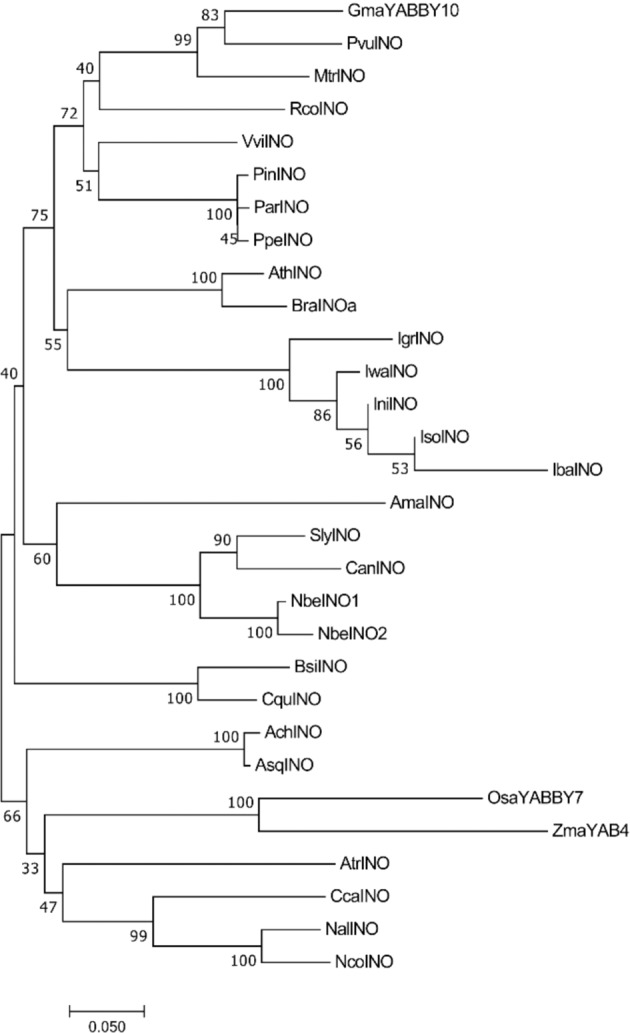
Phylogenetic relationship of INO proteins from grapevine and other plants. A phylogenetic tree was constructed from 30 INO amino acid sequences identified in different species showing an orthologous relationship to INO, including the grapevine predicted full-length INO. Protein nomenclature details are reported in Supplementary Table S1. The phylogenetic tree was built using the Neighbor-Joining method and the p-distance in MEGA7. All positions with <85% site coverage were eliminated. The optimal tree is shown, and bootstrap support is shown next to the branches

Beside Arabidopsis, studies on INO have been extended to further species also with divergent ovule morphology, in the attempt to clarify the evolutionary steps behind ovule development across angiosperms and especially the reduction of integuments number^21–23,25,26^. Comparison of expression domains of *INO* orthologues suggested so far that the role of INO for outer integument growth was established early in angiosperms lineage and is widely conserved, despite some divergences in protein function^12,27^. To explore the relationship of the grape INO with these characterized INO proteins and infer functional information, a phylogenetic tree was built based on 29 full-length INO orthologues together with the grapevine INO (Supplementary Table S4). INO proteins from monocots and basal Angiosperms including Annona species clustered independently, in line with their highest phylogenetic distance. The grapevine INO protein clustered closer to the Arabidopsis protein and INO orthologues from other Rosidae, while INO from Asteridae, including tomato, grouped as a separate clade, independently of the number of integuments found in their ovules (Fig. 2).

### Cloning of *VviINO.1* and *VviINO.2* cDNAs and expression characterization

To further characterize *VviINO*, its coding sequence was isolated from flowers of the grapevine cv. Italia harvested at 10% anthesis. Surprisingly, two cDNA were cloned (*VviINO.1* and *VviINO.2*), 531 and 612 bp long, respectively. Sequence alignment to the grapevine reference genomic sequence showed that the shortest cDNA corresponded to the expected CDS according to the V2 prediction, including six exons and five introns with no polymorphisms and encoding a 176 aa protein. The second cDNA clone corresponded to an incompletely spliced mRNA entirely retaining the intron IV (81 bp). *In silico* translation of this produced a shorter protein (131 aa), due to the presence of an in-frame stop codon. Comparative 3D modeling showed that the 176 aa protein predicted from fully processed mRNA consisted of five small alpha-helices followed by longer unstructured loops, typically observed in regulatory proteins^32^. The removal of the last 45 residues, due to intron retention in the incompletely spliced form, determines the loss of two and a half helices (blue portion, Supplementary Fig. S3), which are predicted to be deeply involved in the stabilization of the interactions with DNA.

**Fig. 3:**
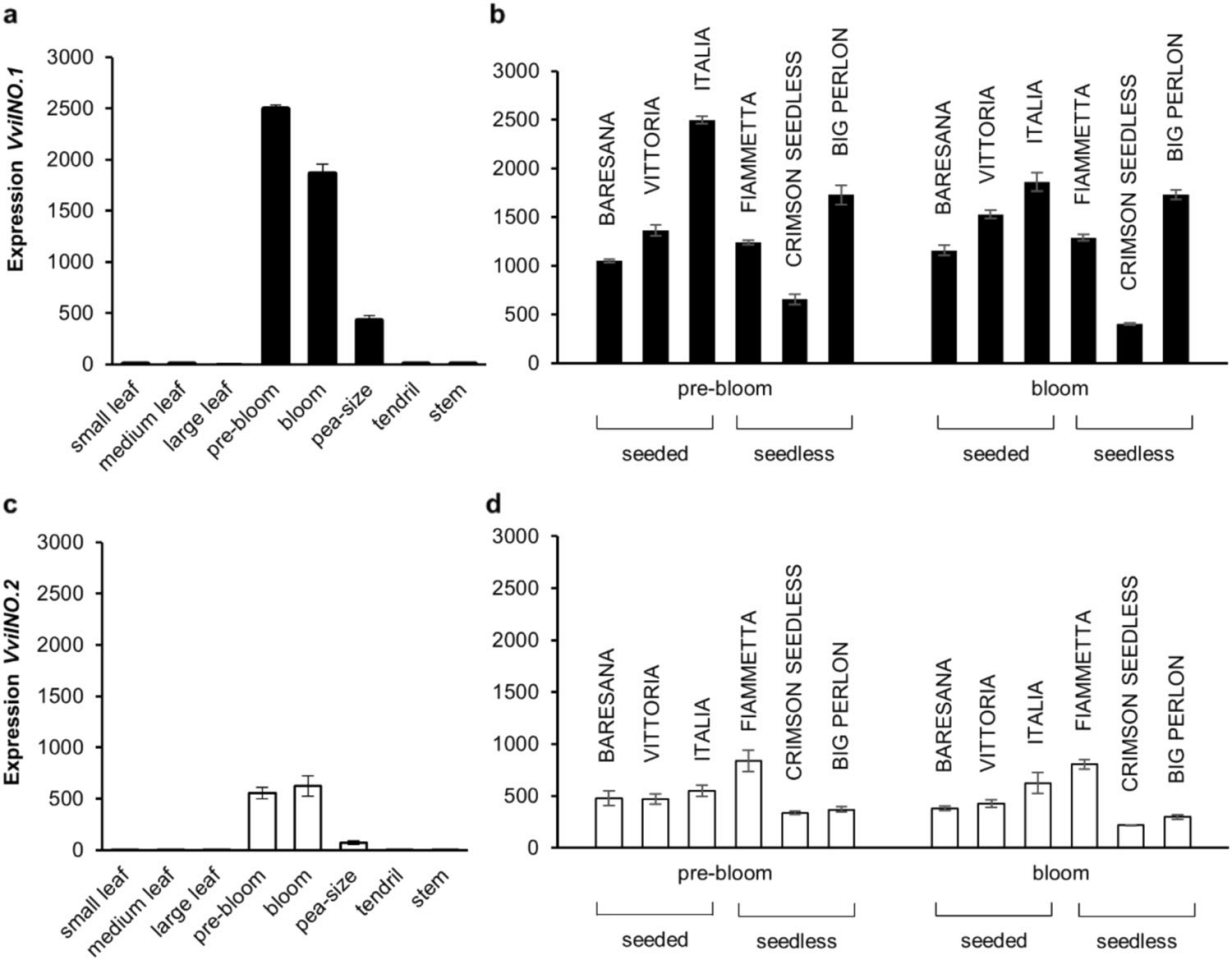
Expression of *VviINO.1* and *VviINO.2* transcripts in grapevine tissues and in flowers of seeded and seedless varieties at pre-bloom and bloom developmental stages. The expression of *VviINO.1* (**a**, **b**) and *VviINO.2* (**c**, **d**) transcripts was quantified using an MGB Taqman-specific assay. Expression was analyzed in different organs at different developmental stages (pre-bloom, bloom, berry pea-size, young leaves, medium leaves, old leaves, tendrils, and stems) in the cultivar Italia (**a** and **b**) or only in flowers at the pre-bloom and bloom stage in additional cultivars with seeds (Baresana, Vittoria) and in seedless cultivars (Fiammetta, Crimson seedless, and Big Perlon) (**b**, **d**). Means are calculated from a technical triplicate of three biological replicates. Error bars show standard errors

We tested the expression of each of the two mRNA in different plant organs by applying specific assays. Both *VviINO.1* and *VviINO.2* were specifically expressed in flowers and young fruits (Fig. 3a, b), as expected for *INO* gene and differently to other *YABBY* family members which were expressed also in other vegetative organs; the only exception was the *VviCRC*, that similarly as *VviINO* mRNAs also showed either low or no expression in vegetative tissues (Supplementary Fig. S4). The highest expression was found at the onset of flowering, while expression decreased at full bloom and in berries at the pea-size stage. However, the incompletely spliced mRNA showed a lower expression compared to the fully processed form (Fig. 3a, c). The accumulation of both *VviINO.1* and *VviINO.2* mRNAs was also compared in flowers at pre-bloom and bloom stages in seeded and seedless cultivars. Samples were collected from three seedless cultivars Big Perlon, Fiammetta, and Crimson seedless, and two additional seeded cultivars Baresana and Vittoria (Fig. 3b, d). No relevant differences in expression levels associated with seedless and seeded cultivars were found, but the incompletely spliced mRNA always consistently showed a lower accumulation compared to the fully processed form.

### Functional analysis of *VviINO.1* and *VviINO.2*

Recent experiments in Solanaceous species demonstrated that, despite the conservation in expression pattern and role, the *SlINO* gene was not able to complement the Arabidopsis *ino-1* mutant^27^. Therefore, we wanted to test whether *VviINO*, differently from *SlINO*, can complement the Arabidopsis mutant.

The Arabidopsis *ino-1* mutant is vegetatively normal, but, as described, lacks the outer integument development on both sides of the ovule primordium and asymmetric growth during ovule development^13,18,33^. At anthesis, the nucellus is covered only by the inner integument, and bending of the chalaza does not progress and the micropyle lay in a line with the funiculus. This mutant was chosen for our cross-species complementation analysis. Both cloned *VviINO.1* and *VviINO.2* CDS were transferred to a segregating Arabidopsis population derived from the mutant, under the control of the *AthINO* promoter, previously described^13,20,34^. After selection of homozygous T3 plants in *ino-1* background, vegetative and ovule phenotypes were observed. No differences in the vegetative growth were observed for any of the six and nine lines selected carrying *VviINO.1* and *VviINO.2*, respectively (Supplementary Fig. S5), even though some variability was observed in the size of silique in mature plants (Supplementary Fig. S6). Careful observation of ovule phenotypes both by stereo and optical microscope showed that both *VviINO.1* and *VviINO.2* coding regions were able to restore outer integument growth. However, differently to complementation with *AthINO* transgene, which leads to the high frequency of full complementation^13,35^, transgenic plants complemented with grape INO CDS exhibited different ovule morphologies consistent inside lines. We have classified lines as “*wild type-like*” when normal ovule development was fully rescued or alternatively as *“sup-like”*, “*weak-ino-like”*, or *“ino-like”* when some atypical growth of outer integument or no growth at all was observed, similarly to^35,36^. To further support our scoring, some lines with representative morphologies were also analyzed by CRYO-SEM (Supplementary Fig. S7).

**Fig. 4:**
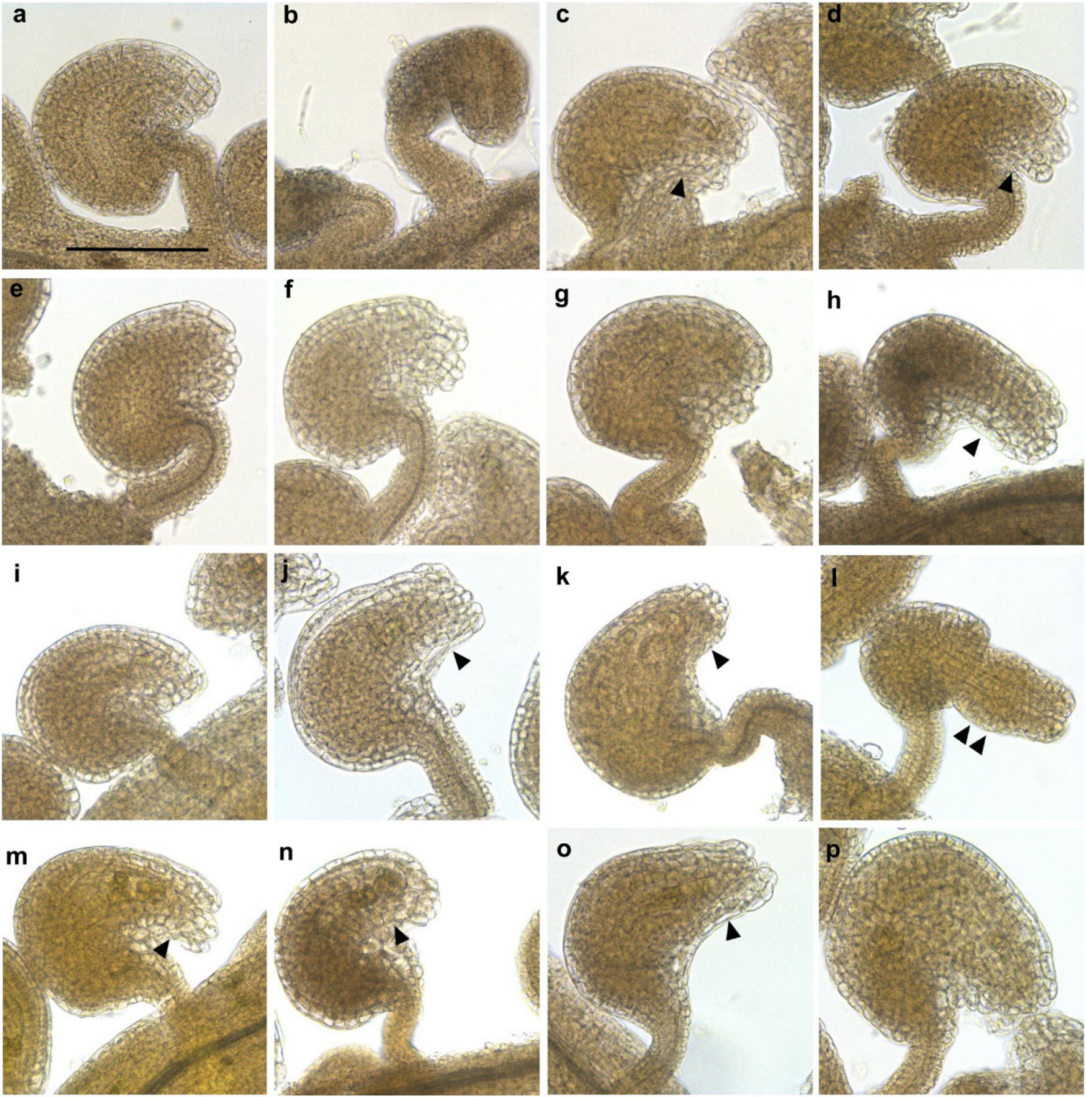
Transgenic complementation experiments of *A. thalianaino-1* by *VviINO.1* and *VviINO.2* under the Arabidopsis promoter. In wild-type ovules (**a**), due to the asymmetric growth of the outer integument, the micropyle lies adjacent to the funiculus. Differently from wild-type, *ino-1* ovules (**b**) typically show no outer integument growth due to the absence of a functional INO protein. The only inner integument is present and micropyle lies in a line from the funiculus. **c**–**h** show representative phenotypes scored in *VviINO.1* transgenic lines (#1, #3, #4, #13, #14, and #15, respectively). Asymmetric growth of the outer integument is always visible in these lines. Some symmetric growth is also visible leading to a *"sup-like"* phenotype (arrows in **c**, **d**, and **h**). **i**–**p** show representative phenotypes scored in *VviINO.2* transgenic lines (#2, #23, #28, #29, #30, #31, #32, and #33, respectively). Asymmetric growth of the outer integument was visible in all lines. Reduced outer integument growth leading to the only partial covering of the inner integument as in the *weak-ino* mutant (allele *ino-4*) can be also observed (**l**, double arrow shows the uncovered inner integument). Symmetric growth of variable importance was often appreciated (arrows in **j**, **k**, **m**, **n**, **o**). Single arrow: outer integument growth from the adaxial side. Double arrow: inner integument uncovered by outer integument. Ovule phenotypes were consistent inside genotypes and representative pictures were chosen for each genotype. All pictures were taken with the same magnification 20x. Bar, in **a**, 100 µm

Observation of transgene effects on *ino-1* ovule morphologies are summarized in Table 1, and representative photos are shown in Fig. 4. Ovules of transgenic lines expressing *VviINO.1* exhibited either wild-type growth (Fig. 4e–g) of the outer integument or a “*sup–like*” phenotype (Fig. 4c, d, h) with outer integument growing symmetrically on both sides of the ovule primordium. This demonstrates that *VviINO.1* can restore the compromised outer integument growth during ovule development and, to some extent, also an asymmetric growth. Surprisingly, all transgenic lines expressing *VviINO.2* rescued the outer integument growth too. In addition to two lines fully recovered to normal ovule morphology (Fig. 4i, p), six lines showed a partial “*sup-like*” phenotype. However, symmetric growth in these lines was much weaker compared to the *sup* mutant (Fig. 4j, k, m–o). Finally, one transgenic line showed a typical “*weak-ino*” phenotype (Fig. 4l).

**Table 1:** Transgenic complementation of *ino-1*

Genotype	Transgene	Line	Ovule phenotype	Seed set/silique	Comparison to *ino-1* (1)	Comparison to wild-type (1)
Wild type	None	*wt*	*wild type-like*	50 ± 5.7	/	/
*ino-1*	None	*ino-1*	*ino-like*	1 ± 1.2	/	/
*ino-1*	*VviINO.1*	#1	*sup-like*	25 ± 3.6	8.83E-19	1.56E-15
		#3	*sup-like*	14 ± 1.3	3.31E-20	4.19E-21
		#4	*wild type-like*	45 ± 3.2	1.72E-26	Not significant
		#13	*wild type-like*	36 ± 3.0	2.05E-24	2.74E-09
		#14	*wild type-like*	50 ± 4.3	8.45E-25	Not significant
		#15	*sup-like*	16 ± 1.8	3.12E-19	2.39E-20
*ino-1*	*VviINO.2*	#2	*wild type-lik*e	40 ± 3.3	1.11E-24	2.51E-06
		#23	*sup-like*	21 ± 5.2	1.45E-13	3.30E-16
		#28	*sup-like*	19 ± 2.2	5.62E-20	9.15E-19
		#29	*weak ino-like*	12 ± 1.5	7.93E-18	1.06E-21
		#30	*sup-like*	35 ± 3.8	4.16E-22	3.53E-09
		#31	*sup-like*	32 ± 5.4	1.36E-17	1.04E-10
		#32	*sup-like*	28 ± 2.5	2.33E-23	5.45E-14
		#33	*wild type-like*	38 ± 2.6	2.18E-26	1.06E-07
		#27	*sup-like*	32 ± 5.6	1.91E-17	3.88E-10

Ovule phenotypes were assessed by flower observation from independent plants with a dissecting stereomicroscope and optical microscope for each transgenic line. The average seed set was measured counting the average number of seeds per silique in four siliques and three independent plants. The seed set was also assessed in wild-type and *ino-1* plants for comparison. (1) A statistical *t* test was conducted to assess the significance of observed differences in seeds

*Ino-1* mutant is strongly affected in female fertility and homozygous plants produce approximately one to three seeds^18^. Thus, the seed set allows additional evaluation of the complementation. In our hands, wild-type plants produced more than 50 seeds on average per silique. Transgenic lines presented a variable number of seeds per silique, but for all the seed set was significantly different compared to the *ino-1* mutant. Only two lines carrying *VviINO.1* showed a seed set comparable to that of wild-type plants. Interestingly, both lines were previously classified as “*wild type-like*” according to their ovule morphology. Seed set in all other lines was significantly different to the wild type, even though also significantly different to *ino-1*, with an average seed number per silique proportional to the rescue of the wild-type ovule morphology scored by microscope (Table 1).

**Fig. 5:**
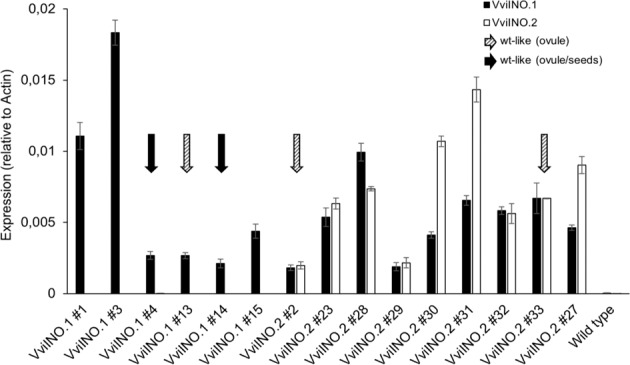
Expression of *VviINO.1* and *VviINO.2* transcripts in flowers of transgenic Arabidopsis lines. Expression levels of *VviINO.1* (black bars) and *VviINO.2* (white bars) transcripts in Arabidopsis flowers were quantified using the Taqman MGB-specific assay. Several pre-bloom flowers were collected for each transgenic line. Flowers of wild-type Arabidopsis plants were included as a control. Relative expression to actin was plotted in the graph. Transgenic lines showing fully complemented phenotype (“*wild type-like”*) either only by microscope ovule observation or also by seed set evaluation are highlighted with dashed and solid arrows, respectively. Transgenic lines with no associated arrow were complemented but showed *"sup-like*" phenotype and lower seed set

Finally, we have investigated the molecular basis of the different levels of complementation. The expression of both transgenes was quantified by using a specific assay (Fig. 5).

As expected, no expression was found in the wild-type Arabidopsis, and lines transformed with *VviINO.1* did not show any expression of *VviINO.2*. On the contrary, lines transformed with *VviINO.2* produced both mRNAs, confirming that the *VviINO.2* is likely an mRNA intermediate that can be successfully further processed in Arabidopsis to encode the complete functional protein, thus explaining the observed rescue in ovule morphology in these lines. Unfortunately, since we only conducted a relative quantification, the expression of *VviINO.1* cannot be compared either to its endogenous expression level in grape nor to the expression of *AthINO*. Relative expression of both transgenes was variable in the different lines, and an inverted relationship between transgene expression and complementation level was observed, which was especially evident in lines carrying the *VviINO.1* transgene.

Altogether these results demonstrate that *VviINO* can restore outer integument growth in *A. thaliana ino-1* mutant as well as a partial asymmetric growth, therefore its function is conserved across the two species.

## Discussion

In this work, we have first of all identified the grapevine orthologue of the *AthINO* transcription factor, starting from the recent characterization of the grapevine *YABBY* gene family^28^. We confirmed that, according to all recent grapevine genome annotations, *VviINO* is encoded by a unique gene located on top of chromosome 1 comprising six exons and five introns. Differing from what expected from current annotations, cDNA cloning resulted in the identification of two mRNAs, the second one retaining the intron four. Alternative splicing due to intron retention could have functional implications, especially when translation results in a mis-functional protein due to a frameshift mutation^37,38^. However, we demonstrated that the *VviINO.2* mRNA can be further spliced, even in a heterologous system, thus suggesting its presence is more likely related to an incomplete processing event or to an mRNA storing mechanism than working through translation in a mis-functional protein, as already reported in the literature for other plant genes^39^. We confirmed a specific expression in flowers and fruit (pea size) while no expression was found in other organs similarly as reported by other authors^28^, in agreement with the specific role of INO in ovule development.

In the past, detailed characterization of *INO* expression patterns in several species suggested wide conservation of INO function down to the early divergent Angiosperm clades^21–24^. Despite that, more recent functional studies provided controversial results. While VIGs silencing of the *NbINO* orthologue in tobacco inhibited the growth of the outer cell layer of the integument, leading to a decrease in both integument extension and ovule curvature, the tomato *SlINO* coding region was not able to complement the Arabidopsis *ino-1* mutant^27^. With the final aim to deepen our knowledge of grapevine reproductive biology and especially ovule development to identify new targets for breeding purposes, we have enquired VviINO protein function conservation. By comparing protein sequences from 30 INO orthologues, we found that VviINO grouped in a clade with AthINO while both were more distantly related to SlINO. This supported the hypothesis that VviINO could complement the Arabidopsis *ino-1* mutant phenotype, unlike SlINO. Results of cross-species complementation demonstrate that the VviINO can indeed successfully functionally complement the Arabidopsis *ino-1* mutant phenotype when expressed from the AthINO promoter. We analyzed the ovules of 6 independent T3 transgenic lines expressing the *VviINO.1* cloned CDS and 9 independent T3 transgenic lines expressing the *VviINO.2* alternative CDS which, as previously mentioned, can be further spliced in Arabidopsis to encode a functional VviINO protein identical to that encoded by the *VviINO.1* transcript. Careful microscopic observations showed that in all lines the outer integument growth and some ovule curvature was rescued. Seed set evaluation also confirmed a significantly different behavior in the transgenic lines compared to the background *ino-1* line. However, differently from Arabidopsis lines complemented with the *AthINO*, we observed a high number of transgenic lines displaying a *"sup*-*like"* phenotype^35^, with also outer integument growth from the adaxial side of the ovule primordium associated with a still partially reduced seed set. This was reminiscent of behaviors previously observed in domain swap experiments. Replacement of the C-terminus of *AthINO* with *AthCRC* resulted in a significant proportion of transgenic lines that contained ovules with a *"sup*-*like"* phenotype, supporting the involvement of this region in the repressive action of SUP^36^. Our results strongly resembled these results, suggesting that, although VviINO can effectively complement the outer integument growth-promotive effects in the *ino-1* mutant, it was less responsive than the endogenous *AthINO* to the SUP inhibition. Interestingly, alignment of VviINO and AthINO protein sequences highlighted low conservation of the C-terminal portion in the grapevine protein (Supplementary Fig. S2), which could explain the less effective SUP suppression and the consequent *"sup*-*like"* phenotype. Furthermore, we speculated that the consequent abaxalization of the adaxial domain would be likely more pronounced in lines with higher transgene expression and that this could likely explain the observed variability in phenotypes. Accordingly, we found a correlation between compromised ovule asymmetric growth and the expression levels of *VviINO.1* and *VviINO.2* transcripts, which further supports our data interpretation (Table 1 and Fig. 5).

These data improve our understanding of grapevine ovule development, with potential implications also for table grape breeding. The major source of seedlessness currently exploited in cultivated grapevine has been recently characterized, being due to an amino acid substitution in the *VviAGL11* gene controlling seed coat development and lignification, downstream of ovule development^11^. Accordingly, no difference in the *VviINO.1* and *VviINO.2* transcripts expression was found in seeded and seedless grape varieties (Fig. 3). However, alternative sources with potential implications for breeding purposes could also exist. The description of seedless normal size fruit production in *Ts* mutant of *A. squamosa* lacking the orthologous *INO* gene^12^, beside the demonstrated functional conservation in ovule development of *VviINO* (this work), supports the *VviINO* gene as a candidate for grapevine seedlessness. However, despite findings in Annona crop, in Arabidopsis *ino-1*, as previously mentioned, “fruit” development appears as compromised^18,19^. The different effect of *ino* defects in ovule development and on seeds and fruit production in Annona and Arabidopsis species has been the subject of further characterizations^12,40^. In these studies, the Arabidopsis *ino-1* pollen tubes grow through the transmitting tract but were never observed inside the micropyle, and the majority of ovules fail to form embryo sacs. In contrast, in the *A. squamosa Ts* mutant, pollen tube growth was more normal often targeting the micropyle, and most of the ovules contained fully developed, but sometimes degenerating, embryo sacs. Authors have speculated that the outer integument role in pollen tube guidance and embryo sac development could have been a recent acquisition in Arabidopsis. They suggested that the absence of an essential role of the outer integument in pollen tube guidance in Annona could be related to its endostomal type of micropyle, with the outer integument not fully covering the inner integument and participating in the micropyle^12,41^. Furthermore, concerning embryo sac development, a higher sensitivity to changes in integument development could be due to a much thinner fraction of tissue around the female germline in the tenuinucellate Arabidopsis compared to the crassinucellate Annona. Interestingly, grapevine also shows an endostomal micropyle and several cell layers surrounding the embryo sac^12,42^. Only comparative studies in grapevine plants knocked-out for the now confirmed grapevine functional VviINO will conclusively allow to define implications of the defect in outer integument development for pollen tube guidance and embryo sac development and thus on seed and fruit production, eventually validating the utility of VviINO for table grape breeding. Interestingly, early genetic studies on grape seedlessness reported that seed coat hardness and endosperm/embryo development were behaving as separate sub-traits, confirming the existence of alternative contributions^43^. Moreover, more recently QTLs studies have revealed the contribution of a region located on top of chromosome 1 including *VviINO* gene to the total seeds fresh weight (TSFW) per berry trait^44^, further supporting *VviINO* as a candidate for grapevine seedlessness. An association to seedlessness in this genomic part of Chr1 was also confirmed by resequencing of seedless and seeded varieties, even though no associated SNP located in this gene were found in the studied panel^10^.

In conclusion, sequence comparison and the rescue of the outer integument growth in all Arabidopsis *ino-1* lines expressing the VviINO protein from the AthINO promoter demonstrate that *VviINO* is the *AthINO* orthologue and that it plays the same function in promoting outer integument growth during ovule development. The high number of transgenic lines displaying a "*sup-like"* phenotype found in our cross-species complementation suggests a reduced sensitivity of VviINO compared to AthINO to the Arabidopsis SUP-mediated repression of expression in the adaxial side of the ovule primordia. Therefore, the mechanism involved in the tight control of INO spatial expression for proper ovule asymmetric growth could have partially diverged in the grapevine. Now that the functional involvement of *VviINO* in outer integument growth during grape ovule development has been demonstrated, functional studies in grape can further elucidate the mechanism for the asymmetric growth and the impacts on fruit and seed formation and their potential implications for table grape breeding purposes.

## Materials and methods

### In silico analysis

BLASTP searches (e-value < 1×e^−5^ and identity >40%) against the PN40024 *Vitis vinifera* genome 12X V2 prediction, available on the CRIBI Biotech website (http://genomes.cribi.unipd.it/)^29^, with protein sequences of the *Arabidopsis thaliana YABBY* family, retrieved from the TAIR database (The Arabidopsis Information Resource, http://www.arabidopsis.org/ gene ID: AT1G08465, AT1G23420, AT1G69180, AT2G26580, AT2G45190, AT4G00180), confirmed the previously identified grapevine *YABBY* genes as the most likely orthologous candidates^28^. Positional and structural information for each member of the family on the latest genome assembly 12X.v2^30^, for the V2 annotation or other available annotations, were downloaded from https://urgi.versailles.inra.fr/Species/Vitis/Annotations. Sequence alignments were performed using the MSA tool MUSCLE in MEGA7 software or at http://www.ebi.ac.uk/Tools/msa/muscle/ using default settings. Grapevine protein sequences corresponding to the V2 prediction were downloaded from the CRIBI website. Only the longest peptide sequence of each gene was used. ngLOC tool (http://genome.unmc.edu/ngLOC/index.html) was applied to predict the subcellular localization of the YABBY proteins. All proteins were submitted to Pfam (http://pfam.xfam.org/search/sequence) to verify the presence of the YABBY domain.

### Orthologous relationship from genetic distances and phylogenetic analysis

To establish orthologous relationships for grapevine *YABBYs* with Arabidopsis, genetic distances and trees were estimated by using the MEGA7. All ambiguous positions were removed for each sequence pair, and the number of base pairs or amino acid differences per site between CDS or protein sequences was used for estimating distance matrices. Orthologous relationships and nomenclature are based on the comparison of the longest CDS with the six Arabidopsis *YABBY* CDS sequences according to rules established by the Grapevine Super Nomenclature Committee^31^. An unrooted UPGMA tree (*p-distance* method) was constructed from CDS distances. Branch tree support values were obtained from 1000 bootstrap replicates and branches with values below 70% were condensed.

The evolutionary history of INO proteins identified in different species was inferred using the Neighbor-Joining method in MEGA7. The evolutionary distances were computed using the *p-distance* method and are in the units of the number of amino acid differences per site. The analysis involved 30 amino acid sequences (Supplementary Table S4). All positions with less than 85% site coverage were eliminated. The optimal tree is shown and the percentage of replicate trees in which the associated taxa clustered together in the bootstrap test (1000 replicates) are shown next to the branches.

### Comparative 3D modeling

The 3D comparative model of the wild-type VviINO.1 protein (protein length 176 aa) was prepared by performing a multi-template modeling session by using Modeler^45^. 3D crystallized structures used for the multi-template modeling, predicted by using fold recognition methods analysis were 2lef.pdb and 3cmv.pdb showing 30% of identical amino acids with VviINO.1 sequence^46^. The 3D comparative model of the shorter VviINO.2 isoform (protein length 131 aa) was built by removing the last 45 residues by using PyMOL. For the modeling of the protein–DNA complex, the obtained VviINO.1 3D comparative model was superimposed to 2lef.pdb for docking the DNA molecule (duplicated from 2lef.pdb) within the VviINO.1/2 3D comparative models.

### Plant materials

For expression analysis, samples from stems, tendrils, flowers, berries, and leaves were collected from the *V. vinifera* cv. Italia from an experimental vineyard located in Valenzano, Bari (Italy). In details, flowers were collected at the pre-bloom stage corresponding to 10% caps off (E-L 19) (Coombe, 1995) and at the bloom stage of 50% caps off (E-L 23); berries were sampled at the pea-size stage of 7-mm diameter (E-L 31); small leaves were collected when the shoot bear was approximatively five separated leaves (E-L 12), medium leaves corresponded to 16 separated leaves (E-L 19), and large leaves represented leaves before senescence (E-L 31). For the expression profiles in seedless and seeded cultivars flowers from the same developmental stages of pre-bloom and bloom as previously described were collected also from the variety Big Perlon, located in the same experimental field as Italia, and the varieties Baresana, Vittoria, Fiammetta, and Crimson seedless from an experimental field located in Adelfia, Bari (Italy). Three biological replicates were independently collected for each tissue/stage. For functional analysis, the previously described mutant *ino-1* (CS3881) of *A. thaliana* (Landsberg *erecta*)^13^ and the corresponding wild type were used. Since the mutant produces few seeds it is maintained in heterozygous status. All plants were grown in a growth chamber under controlled conditions (8-h light/16-h dark photoperiod, 24 °C/21.5 °C, 70% relative humidity) and watered weekly. The light was provided by warm white fluorescent tubes, 120 to 160 µmolphotons m^−2^ s^−2^.

For expression studies in Arabidopsis, several pre-bloom flowers (up to 100 mg) were collected from different plants belonging to the same homozygous T3 line.

### Expression studies

The total RNA was extracted from grapevine tissues (400 mg for berries and 100 mg for other tissues) or Arabidopsis flowers (100 mg) using Spectrum^TM^ Plant Total RNA kit (Sigma-Aldrich, St. Louis, MO) following the manufacturer’s instructions. To remove genomic DNA, an optional on-column step with RNase-Free DNase I Set (Qiagen, Hilden, Germany) was included immediately after the binding step. After extraction, RNA was further purified in 3 M LiCl (Sigma-Aldrich), precipitated at 4 °C overnight, and centrifuged at 15,000 × *g* for 20 min at 4 °C. The pellet was rinsed with 70% cold ethanol, centrifuged at 13,000 × *g* for 5 min, and then eluted in 50 µl of nuclease-free water.

The first-strand cDNA for quantitative real-time PCR was synthesized with Superscript^®^ III First-Strand Synthesis System (Invitrogen, Carlsbad, USA) starting from 1 µg of RNA and primed using the Oligo (dT)_20_ following the manufacturer’s instructions. cDNAs were diluted ten times in pure water. Quantitative RT-PCR of *YABBY* genes was conducted as described by Symons et al.^47^ in triplicate for each sample using 3 µl cDNA in 1× SYBR Select Master Mix (Applied Biosystems, Foster City, USA) and with 0.4 µM of forward and reverse primer in a total volume of 20 µl. Gene-specific primer pairs were designed with OligoExplorer 1.1.2 avoiding regions of cross-homology for each gene and, for normalization of cDNA levels, for *Actin2* from grape (Supplementary Table S5). CFX96™ Real-Time PCR Detection System (Bio-rad, Hercules, USA) was used, and the data were analyzed with CFX Manager™ software. Copy number for each *YABBY* gene was assessed according to Bottcher et al.^48^, and reaction specificity was confirmed by melt curve analysis, by agarose gel, and by sequencing (Macrogen, Meibergrdeef, The Netherlands).

To measure the expression levels of the two mRNA Vv*iINO.1 and VviINO.2*, we developed TaqMan™ Gene Expression Assays specific for each of the forms, which makes use of a pair of unlabeled PCR primers and the TaqMan probes with a FAM™ dye label on the 5'-end and a minor groove binder (MGB) and a non-fluorescent quencher (NFQ) on the 3'-end (Supplementary Table S5). The cDNA levels were normalized with TaqMan™ Gene Expression Assays for *VviActin2* or *AthActin2* in grapevine and Arabidopsis, respectively (Supplementary Table S5). Real-time PCR conditions were as suggested by the assay and Real-Time PCR Detection System (BioRad, USA). In grape, copy number was estimated in tissues/organs or varieties as previously indicated. Relative quantification to actin was calculated instead for expression analysis in Arabidopsis flowers from transgenic lines.

### DNA constructs and plants transformation

The grapevine *VviINO.1* (531 bp) and *VviINO.2* (612 bp) cDNAs were isolated from *V. vinifera* cv. Italia flowers at the pre-bloom stage by RT-PCR using primers *VviINO-1 forward* and *VviINO-4 reverse* (Supplementary Table S5) and cloned into pJET 1.2/blunt cloning vector system (Thermo Fisher Scientific). Several clones were randomly chosen and sequenced in order to verify the presence of the alternative forms. Both cDNA coding regions were modified by PCR using primers *BamVviINOF* containing a BamHI site and *XbaVviINOR* (Supplementary Table S5) containing an XbaI site. BamHI/XbaI fragments were used to replace the Arabidopsis INO cDNA into the previously described pRJM33 chimera vector carrying the Arabidopsis INO cDNA flanked by the corresponding 5'- (2.3 kb) and 3'- (2 kb) genomic regions^13^. These regions were previously shown to be sufficient to enable complementation of the *ino-1* mutant phenotype^20,35^. The new Arabidopsis genomic::grape cDNA chimera was inserted as NotI fragment into the pMLBART plant transformation vector^49^ to obtain pIM4 and pIM5 plasmids and transferred into the *Agrobacterium tumefaciens* GV3101 strain.

To evaluate the competence of *VviINO.1* and *VviINO.2* to complement the *ino-1* phenotype in *A. thaliana* pIM4 and pIM5 constructs were transformed into an Arabidopsis segregating population for the *ino-1* mutant previously indicated by the Agrobacterium-mediated floral dip method. Transformants were selected by germinating seeds on Murashige and Skoog (MS) medium, 3% (w/v) sucrose, 0.8% (w/v) agar, and 10 μg ml^–1^ phosphinothricin (BASTA). The presence of the transgene was confirmed by PCR using the primers *4CKfor* and *4CKrev* (Supplementary Table S5) that amplify different sized fragments from *VviINO.1* and *VviINO.2* transgenes. The *ino-1* homozygous background was selected genotyping by PCR amplification and sequencing the *A. thaliana INO* gene using primers *ino-1-genfor* and *ino-1-genrev* (Supplementary Table S5). T3 homozygous lines for each of the two transgenes were then selected.

### Optical, stereo, and CRYO-SEM microscopy

Complemented Arabidopsis plants were grown under long-day conditions until flower bud formation and flowers sampled. For microscope observation, samples were prepared immediately before use from flowers fixed in FAA solution (3.7% formaldehyde, 5% acetic acid, 50% ethanol) or from isolated ovaries dissected and stored in 70% ethanol at room temperature. Ovule phenotypes evaluation was performed using bright-field optical microscopy or in dark-field under a stereomicroscope for all transgenic lines in *ino-1* background as well as *ino-*1 and wild-type plants. At least four independent flowers were observed for each line by dissecting stereomicroscope, and pictures were taken by bright-field optical microscopy. Representative lines of each phenotypic class were fixed and prepared for CRYO-Scanning Electron Microscopy (CRYO-SEM). Before observation, the ovaries were rinsed in water, opened with a fine needle and tweezers in order to take the ovules that were immediately observed under CRYO-SEM.

### Seed set evaluation

The average number of seeds per silique was estimated by counting seeds from four siliques per plant and three plants per each line and a t test was applied to evaluate significant differences.

## Supplementary information

Supplementary information

## Data Availability

All DNA and protein sequences used in the paper are available at GeneBank, NCBI, TAIR, repository with indicated accession number/gene ID or at https://urgi.versailles.inra.fr/Species/Vitis and www.cribi.unipd.it.
