# Supplementary material for: Functional conservation of the grapevine candidate gene INNER NO OUTER for ovule development and seed formation
Source: Hortic Res. 2021 Feb 1;8:29. doi: 10.1038/s41438-021-00467-5 (PMC7848007; doi:10.1038/s41438-021-00467-5)
Supplement: Supplementary file 1 — Supplementary information [file 41438_2021_467_MOESM1_ESM.docx]

**Supplementary Information**

**Title: Functional conservation of the grapevine candidate gene *INNER NO OUTER* for ovule development and seed formation**

**Running title: Functional characterization of grapevine *INNER NO OUTER***

Valentina di Rienzo^1,2#^, Zahra Imanifard^3#^, Isabella Mascio^1^, Charles S. Gasser^4^, Debra J Skinner^4^, Ciro Leonardo Pierri^5,6^, Martina Marini^3^, Valentina Fanelli^1^, Wilma Sabetta^7^, Cinzia Montemurro^1,2,8*^, Diana Bellin^3*^


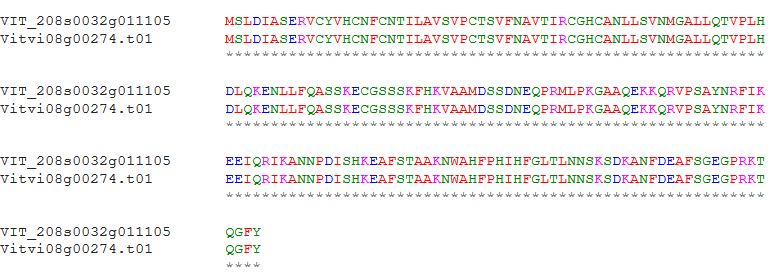


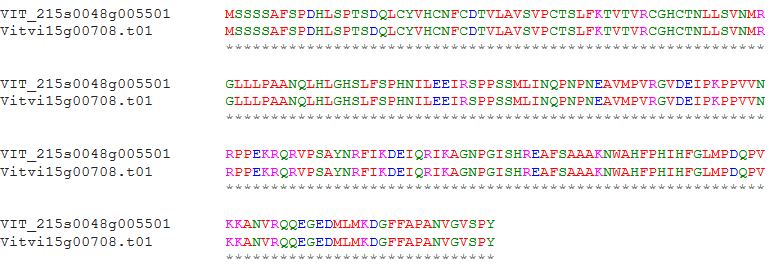


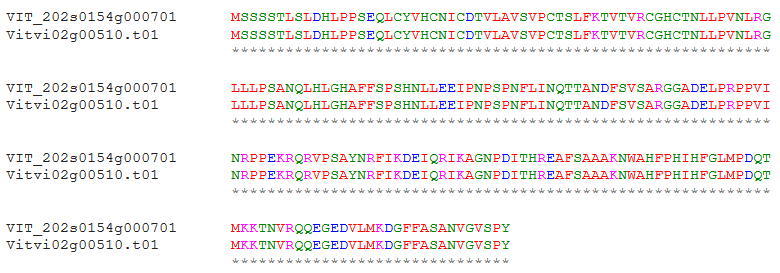


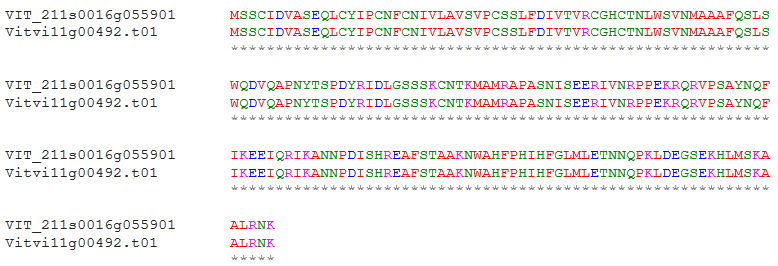


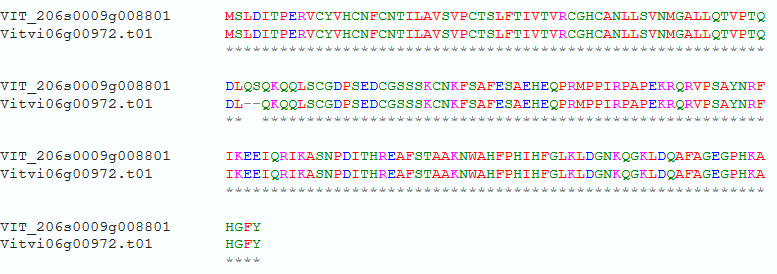


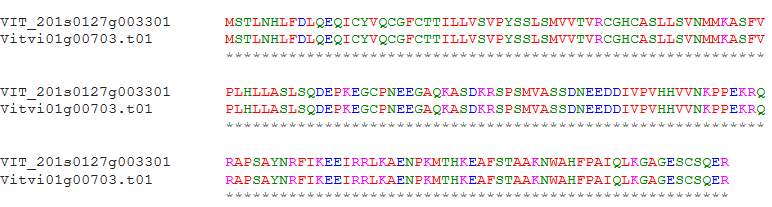


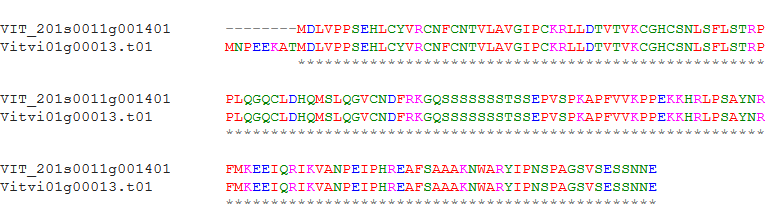


**Figure S1. Pairwise alignments of grapevine YABBY predicted proteins according to the different grape annotations**


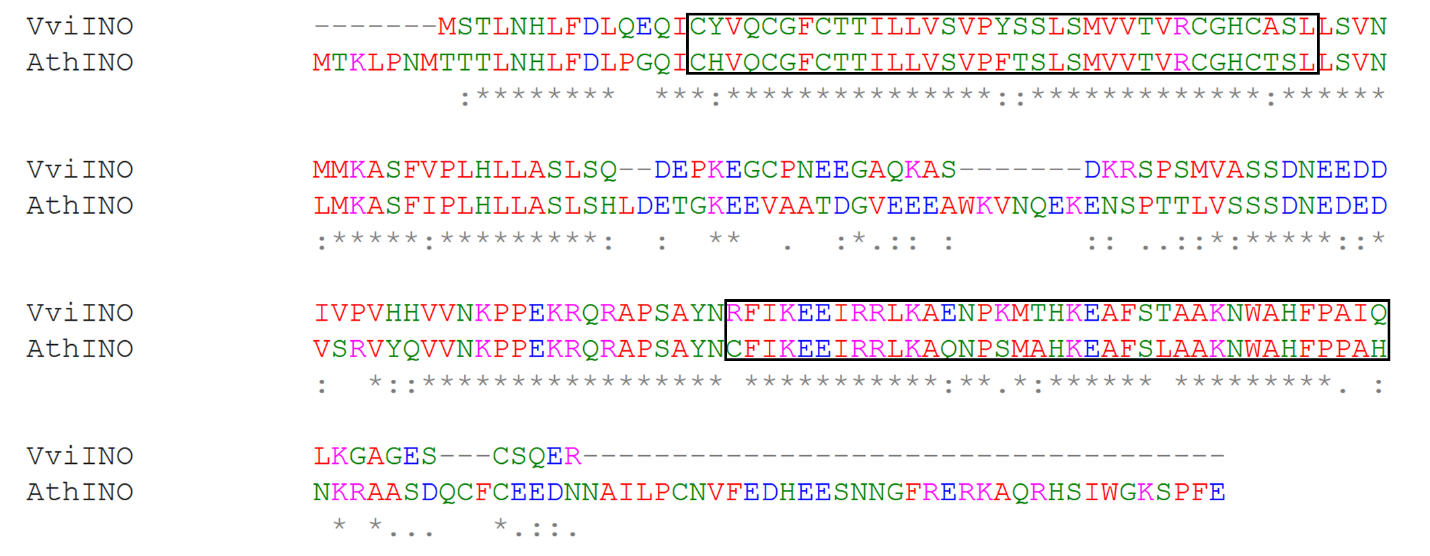


**Figure S2. Amino acids residues conservation among the *V. vinifera* and *A. thaliana* INO proteins.** The grapevine and Arabidopsis INO protein sequences were aligned by Clustal Omega. Conserved amino acids residues between the grapevine and the *A. thaliana* INO deduced proteins are indicated by asterisks. The conserved residues of the putative zinc finger (toward the N-ter) and YABBY (toward the C-ter) domains in agreement with domain description as in Villanueva et al., 1999 are highlighted in a box.

**
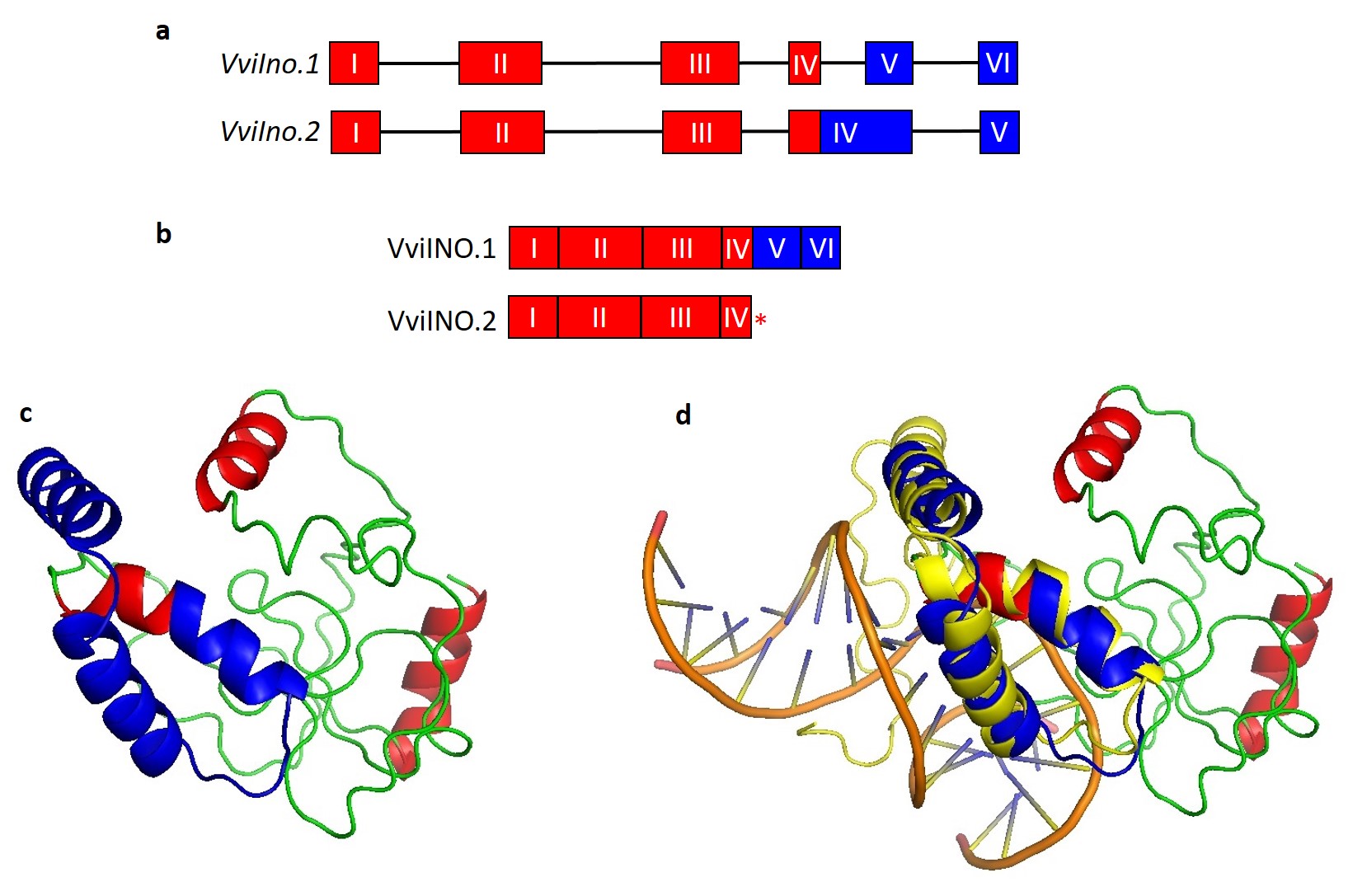
Figure S3. *VviINO.1* and *VviINO.2* annotations and predicted CDS and proteins with 3D comparative models.** (a) Annotations and (b) predicted CDS for each grapevine cloned cDNA. (c) VviINO.1 3D comparative protein model in red, green and blue cartoon representation. The blue region represents the portion lost in VviINO.2. (d) Superimposition of VviINO.1/2 3D models with the C-terminal domain of the transcription factor 2lef.pdb (yellow) interacting with a DNA molecule (orange coil and yellow/blue sticks).

**
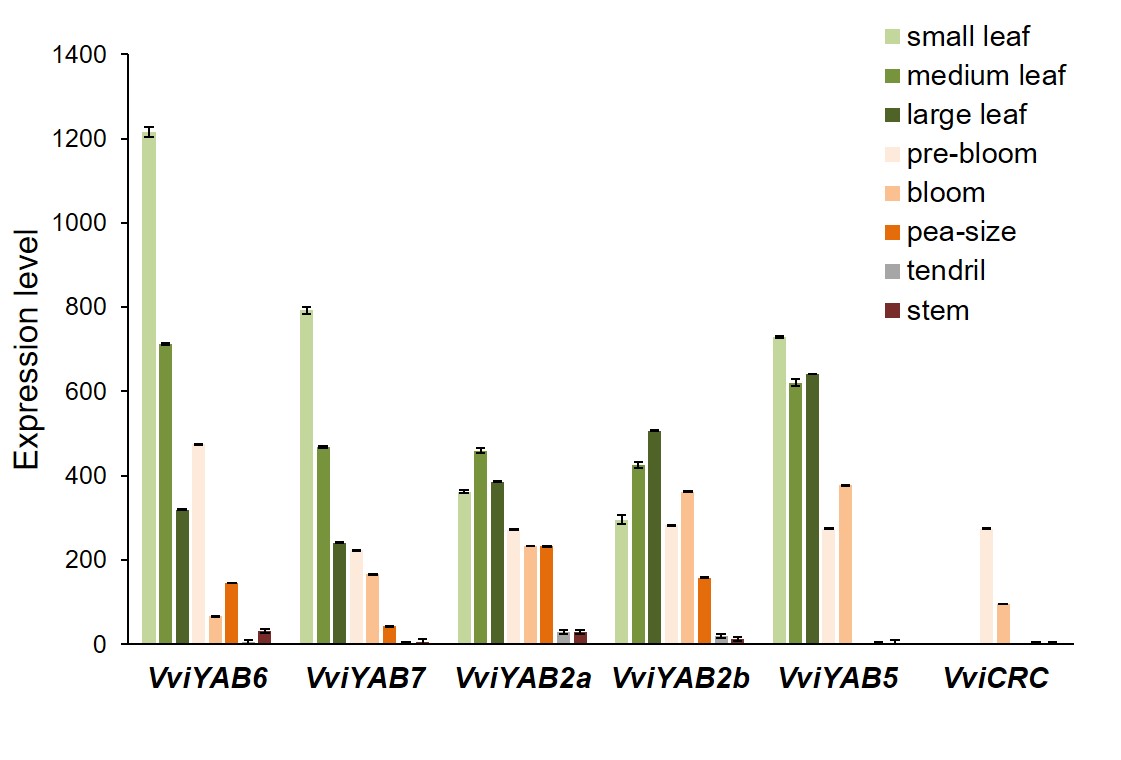
Figure S4.** **Expression of grapevine YABBY genes in tissues of different organs.** Expression was analysed in tissues of different organs collected at different developmental stages from the grapevine cultivar Italia. Absolute expression was quantified by RT-PCR analysis and average values from technical and biological replicates are plotted.

**
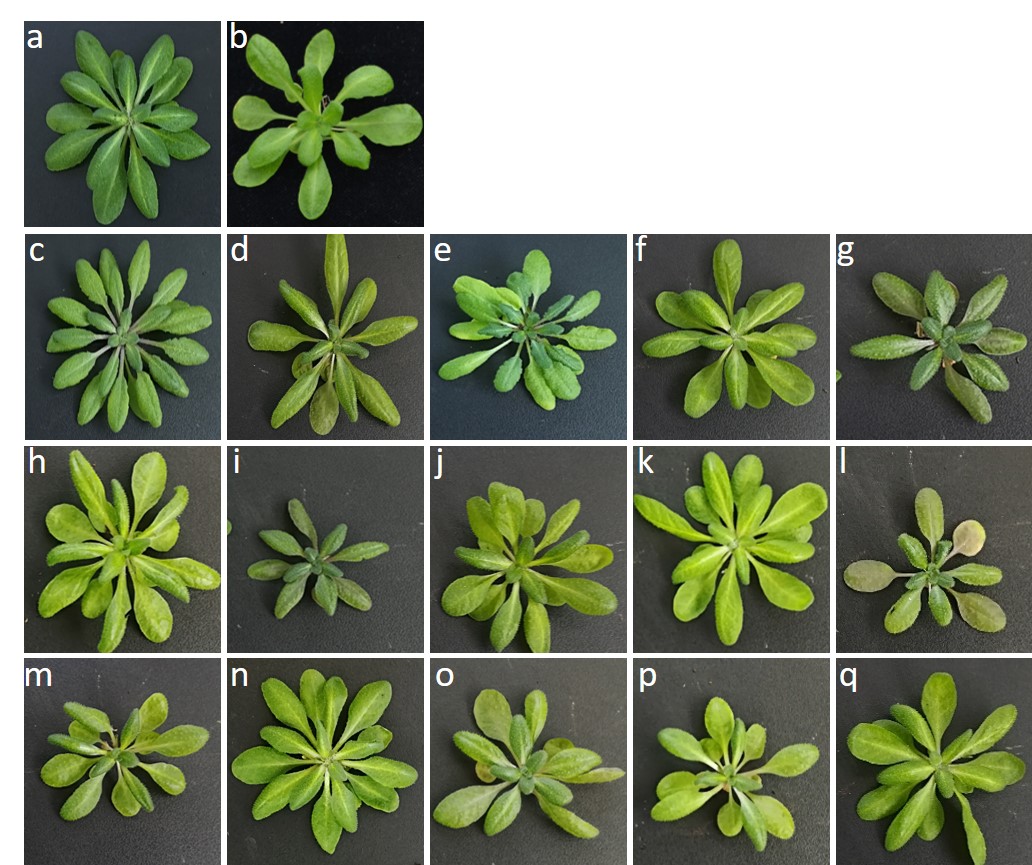
**

**Figure S5.** **Vegetative rosette phenotypes of transgenic Arabidopsis lines expressing *VviINO.1* or *VviINO.2.*** Rosette phenotypes of wild type and *ino-1* mutant plants are shown in (a) and (b). Rosette of each transgenic line expressing VviINO.1 [lines #1 (c), #3 (d), #4 (e), #13 (f), #14 (g) and #15 (h)] or VviINO.2 [lines #2 (i), #23 (j), #28 (k), #29 (l), #30 (m), #31 (n), #32 (o), #33 (p) and #27 (q)] are shown.

**
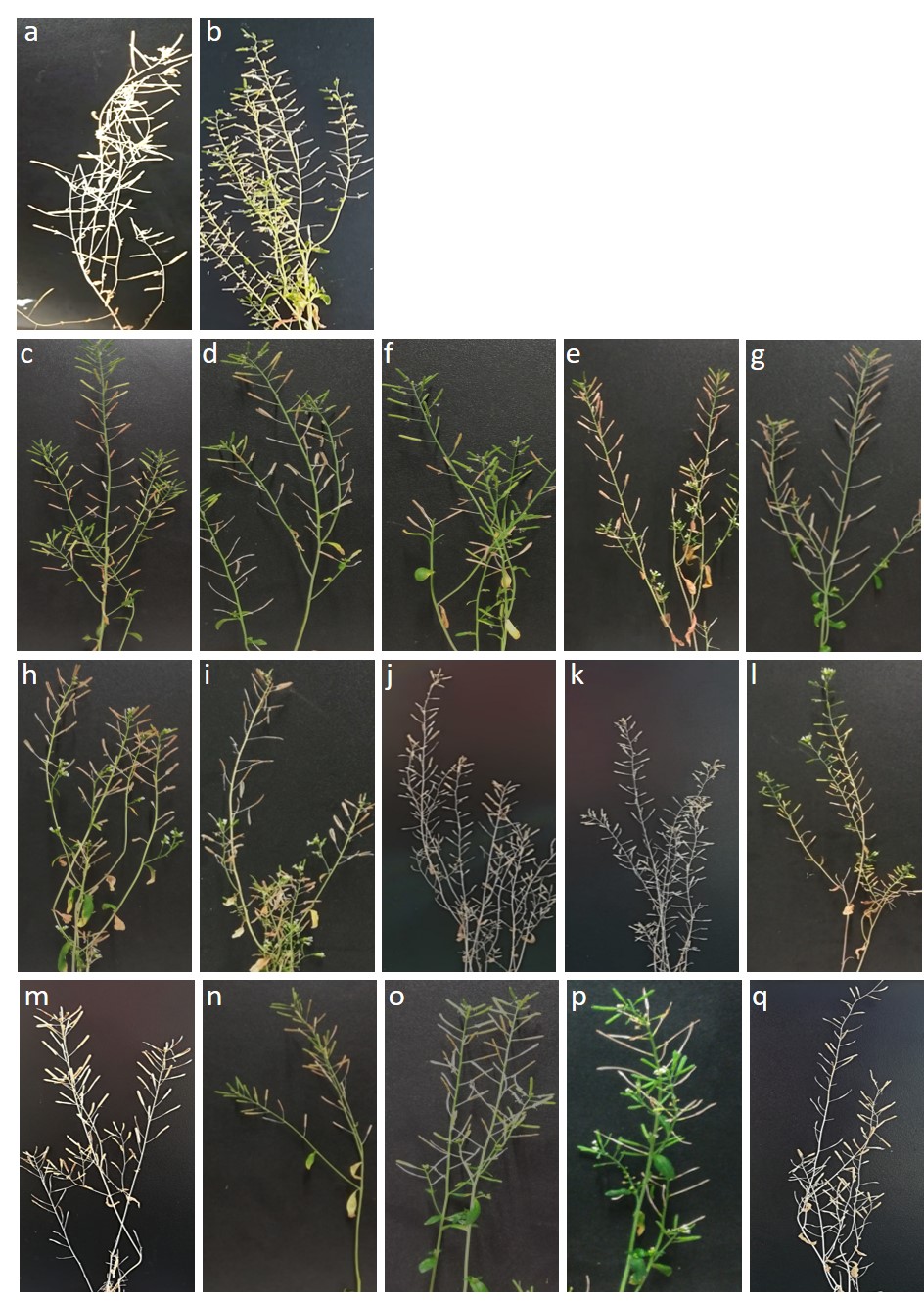
**

**Figure S6. Mature plant phenotypes of transgenic Arabidopsis lines expressing *VviINO.1* or *VviINO.2*** Mature plant phenotypes of wild type and ino-1 mutant plants are shown in (a) and (b). Plants for each transgenic line expressing VviINO.1 [lines #1 (c), #3 (d), #4 (e), #13 (f), #14 (g) and #15 (h)] or VviINO.2 [lines #2 (i), #23 (j), #28 (k), #29 (l), #30 (m), #31 (n), #32 (o), #33 (p) and #27 (q)] were also photographed.

**
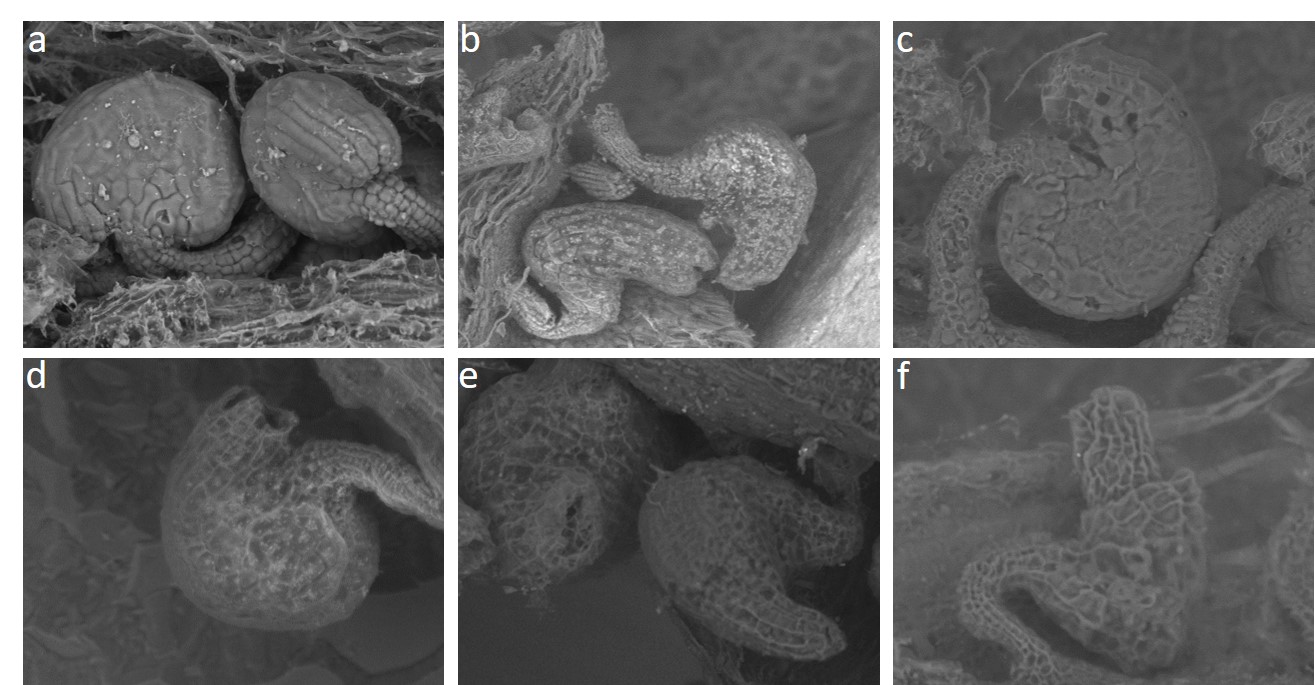
**

**Figure S7. CRYO-SEM observation of transgenic Arabidopsis *ino-1* complemented by *VviINO.1* or *VviINO.2*** Wild type ovule phenotypes (a) were compared to ovule phenotypes of transgenic *VviINO.1* [line #1 (b), line #4 (c), and line #13 (d)] or to ovule phenotypes of transgenic *VviINO.2* [lines #30 (e) and line #29 (f)] by CRYO-SEM.

| **gene_name** | **Annotation** | **Gene_ID(*)** | **chr(*)** | **start (bp)(*)** | **end(bp)(*)** | **gene_lenght(bp)(*)** | **associated_ transcripts(*)** | **exons(*)(**)** | **introns(*)(**)** | **protein_lenght(aa)(*)(**)** |
| --- | --- | --- | --- | --- | --- | --- | --- | --- | --- | --- |
| ***VviYAB2a*** | RefSeq | LOC100256505 | 6 | 13075928 | 13083019 | 7092 | 4 | 6 | 5 | 184(183,182,181) |
|  | V2 CRIBI | VIT_206s0009g00880 | 6 | 13075800 | 13083092 | 7293 | 4 | 6(5) | 5(4) | 184(183,151,150) |
|  | vCost.v3 | Vitvi06g00972 | 6 | 13075928 | 13083019 | 7092 | 4 | 6 | 5 | 184(183,182,181) |
| ***VviYAB2b*** | RefSeq | LOC100267536 | 8 | 5502500 | 5509515 | 7016 | 5 | 6 | 5 | 184(183,182) |
|  | V2 CRIBI | VIT_208s0032g01110 | 8 | 5502499 | 5509606 | 7108 | 9 | 6(5) | 5(4) | 184(183,182,135,134,133) |
|  | vCost.v3 | Vitvi08g00274 | 8 | 5502500 | 5509517 | 7018 | 1 | 6 | 5 | 184 |
| ***VviYAB5*** | RefSeq | LOC100254328 | 11 | 5013892 | 5017654 | 3763 | 1 | 7 | 6 | 185 |
|  | V2 CRIBI | VIT_211s0016g05590 | 11 | 5013767 | 5017918 | 4152 | 2 | 7(5) | 6(4) | 185(151) |
|  | vCost.v3 | Vitvi11g00492 | 11 | 5013892 | 5017654 | 3763 | 1 | 7 | 6 | 185 |
| ***VviYAB6*** | RefSeq | LOC100267708 | 2 | 4804770 | 4807571 | 2802 | 1 | 7 | 6 | 211 |
|  | V2 CRIBI | VIT_202s0154g00070 | 2 | 4804748 | 4807679 | 2932 | 3 | 7(5) | 6(4) | 211(171) |
|  | vCost.v3 | Vitvi02g00510 | 2 | 4804770 | 4807571 | 2802 | 1 | 7 | 6 | 211 |
| ***VviYAB7*** | RefSeq | LOC100243667 | 15 | 14674807 | 14677850 | 3044 | 2 | 7 | 6 | 211(210) |
|  | V2 CRIBI | VIT_215s0048g00550 | 15 | 14674632 | 14678209 | 3578 | 2 | 7(5) | 6(4) | 210(175) |
|  | vCost.v3 | Vitvi15g00708 | 15 | 14675136 | 14677631 | 2496 | 1 | 7 | 6 | 210 |
| ***VviINO*** | RefSeq | LOC100258462 | 1 | 8084464 | 8087242 | 2779 | 2 | 7 | 6 | 179(178) |
|  | V2 CRIBI | VIT_201s0127g00330 | 1 | 8084547 | 8085693 | 1147 | 1 | 6 | 5 | 176 |
|  | vCost.v3 | Vitvi01g00703 | 1 | 8084547 | 8085693 | 1147 | 1 | 6 | 5 | 176 |
| ***VviCRC*** | RefSeq | LOC100261225 | 1 | 237169 | 239545 | 2377 | 2 | 7 | 6 | 175(168) |
|  | V2 CRIBI | VIT_201s0011g00140 | 1 | 237202 | 239334 | 2133 | 1 | 7 | 6 | 160 |
|  | vCost.v3 | Vitvi01g00013 | 1 | 237562 | 239297 | 1736 | 1 | 7 | 6 | 168 |

**Table S1. Structural information for grapevine YABBY family member according to different annotations.**

(*) structural information for each annotation including chromosome (chr), gene start and end positions in basepairs (bp) information on transcripts as well as protein lenght in aminoacids (aa) refer to the latest genome assembly 12X.v2 and were retrieved from <https://urgi.versailles.inra.fr/Species/Vitis/Annotations>

(**) exons/introns numbers and protein lenght in aminoacids in brackets refers to alternative shorter transcripts isoforms. Longest isoform data are reported as main data out of brackets

**Table S2. Localization prediction (a) and domain annotation (b) of grapevine YABBY proteins.**

| **(a)** | |  | | |  |
| --- | --- | --- | --- | --- | --- |
| **Gene ID ^(a)^** | **ngLOC prediction^(b)^** | | **ngLOC prob^(b)^** |  |  |
| VIT_206s0009g00880 | NUC | | 85.85 |  |  |
| VIT_208s0032g01110 | NUC | | 79.86 |  |  |
| VIT_211s0016g05590 | NUC | | 79.37 |  |  |
| VIT_202s0154g00070 | NUC | | 78.55 |  |  |
| VIT_215s0048g00550 | NUC | | 80.33 |  |  |
| VIT_201s0127g00330 | NUC | | 80.16 |  |  |
| VIT_201s0011g00140 | NUC | | 34.91 |  |  |
| (a) Predicted protein sequence corresponding to the longest V2 transcript was selected for each gene | | | | | |
| (b) Localization prediction (prediction) and associated percentage probability of prediction (prob) estimated by nLOC tool (http://genome.unmc.edu/ngLOC/index.html). Nucleus (NUC). | | | | | |

| **(b)** |  | | |  | | |  | |  | |  | |  | |  | |  |  |  |  |
| --- | --- | --- | --- | --- | --- | --- | --- | --- | --- | --- | --- | --- | --- | --- | --- | --- | --- | --- | --- | --- |
| **Gene ID ^(a)^** | **Alignment start** | **Alignment end** | **Envelope start** | | **Envelope end** | **HMM accession #** | | **HMM name** | | **HMM start** | **HMM end** | **HMM length** | **Bit score** | **E-value** | |  |  |  |  |  |
| VIT_206s0009g00880 | 6 | 158 | 5 | | 158 | PF04690 | | YABBY | | 2 | 162 | 162 | 242.6 | 2.80E-72 | |  |  |  |  |  |
| VIT_208s0032g01110 | 6 | 156 | 5 | | 156 | PF04690 | | YABBY | | 2 | 162 | 162 | 232.3 | 4.20E-69 | |  |  |  |  |  |
| VIT_211s0016g05590 | 8 | 158 | 7 | | 158 | PF04690 | | YABBY | | 2 | 162 | 162 | 235.7 | 3.80E-70 | |  |  |  |  |  |
| VIT_202s0154g00070 | 14 | 175 | 13 | | 175 | PF04690 | | YABBY | | 2 | 162 | 162 | 234.9 | 6.40E-70 | |  |  |  |  |  |
| VIT_215s0048g00550 | 15 | 174 | 14 | | 174 | PF04690 | | YABBY | | 2 | 162 | 162 | 239.4 | 2.60E-71 | |  |  |  |  |  |
| VIT_201s0127g00330 | 10 | 165 | 9 | | 167 | PF04690 | | YABBY | | 2 | 160 | 162 | 220.5 | 1.80E-65 | |  |  |  |  |  |
| VIT_201s0011g00140 | 6 | 144 | 5 | | 151 | PF04690 | | YABBY | | 2 | 155 | 162 | 185.7 | 8.90E-55 | |  |  |  |  |  |
| (a) Predicted protein sequence corresponding to the longest V2 transcript was selected for each gene | | | | | | | | | | |  | |  | |  | |  |  |  |  |

**Table S3. Distance matrix for grapevine and Arabidopsis YABBY nucleotide (a) or aminoacids sequences (b)**

| _(a)_ |  |  |  |  |  |  |  |  |  |  |  |  |  |
| --- | --- | --- | --- | --- | --- | --- | --- | --- | --- | --- | --- | --- | --- |
| _VIT_206s0009g00880-VviYAB2a_ |  | _0.019_ | _0.023_ | _0.024_ | _0.024_ | _0.023_ | _0.025_ | _0.022_ | _0.024_ | _0.024_ | _0.023_ | _0.024_ | _0.024_ |
| _VIT_208s0032g01110-VviYAB2b_ | _0.192_ |  | _0.023_ | _0.023_ | _0.024_ | _0.023_ | _0.025_ | _0.022_ | _0.024_ | _0.024_ | _0.024_ | _0.024_ | _0.024_ |
| _VIT_201s0127g00330-VviINO_ | _0.371_ | _0.389_ |  | _0.023_ | _0.022_ | _0.023_ | _0.024_ | _0.023_ | _0.021_ | _0.024_ | _0.023_ | _0.023_ | _0.023_ |
| _VIT_215s0048g00550-VviYAB7_ | _0.347_ | _0.352_ | _0.379_ |  | _0.019_ | _0.023_ | _0.024_ | _0.024_ | _0.024_ | _0.024_ | _0.023_ | _0.022_ | _0.021_ |
| _VIT_202s0154g00070-VviYAB6_ | _0.347_ | _0.341_ | _0.355_ | _0.181_ |  | _0.024_ | _0.025_ | _0.024_ | _0.024_ | _0.024_ | _0.022_ | _0.021_ | _0.022_ |
| _VIT_211s0016g05590-VviYAB5_ | _0.315_ | _0.320_ | _0.360_ | _0.317_ | _0.341_ |  | _0.024_ | _0.024_ | _0.023_ | _0.025_ | _0.019_ | _0.024_ | _0.025_ |
| _VIT_201s0011g00140-VviCRC_ | _0.392_ | _0.392_ | _0.464_ | _0.373_ | _0.384_ | _0.376_ |  | _0.023_ | _0.024_ | _0.023_ | _0.025_ | _0.025_ | _0.024_ |
| _AT1G08465-AthYAB2_ | _0.227_ | _0.283_ | _0.363_ | _0.352_ | _0.363_ | _0.333_ | _0.379_ |  | _0.023_ | _0.025_ | _0.023_ | _0.025_ | _0.025_ |
| _AT1G23420-AthINO_ | _0.384_ | _0.416_ | _0.229_ | _0.416_ | _0.416_ | _0.400_ | _0.451_ | _0.368_ |  | _0.025_ | _0.023_ | _0.024_ | _0.025_ |
| _AT1G69180-AthCRC_ | _0.421_ | _0.443_ | _0.445_ | _0.379_ | _0.392_ | _0.424_ | _0.280_ | _0.429_ | _0.448_ |  | _0.024_ | _0.025_ | _0.024_ |
| _AT2G26580-AthYAB5_ | _0.312_ | _0.355_ | _0.381_ | _0.325_ | _0.339_ | _0.179_ | _0.387_ | _0.328_ | _0.405_ | _0.400_ |  | _0.024_ | _0.024_ |
| _AT2G45190-AthYAB1(AFOFIL)_ | _0.336_ | _0.336_ | _0.365_ | _0.253_ | _0.269_ | _0.325_ | _0.365_ | _0.344_ | _0.413_ | _0.379_ | _0.333_ |  | _0.022_ |
| _AT4G00180-AthYAB3_ | _0.339_ | _0.336_ | _0.371_ | _0.245_ | _0.277_ | _0.328_ | _0.381_ | _0.349_ | _0.405_ | _0.381_ | _0.325_ | _0.277_ |  |
|  |  |  |  |  |  |  |  |  |  |  |  |  |  |
| _(b)_ |  |  |  |  |  |  |  |  |  |  |  |  |  |
| _VIT_206s0009g00880-VviYAB2a_ |  | _0.033_ | _0.041_ | _0.041_ | _0.039_ | _0.038_ | _0.041_ | _0.035_ | _0.042_ | _0.042_ | _0.039_ | _0.040_ | _0.039_ |
| _VIT_208s0032g01110-VviYAB2b_ | _0.180_ |  | _0.042_ | _0.041_ | _0.041_ | _0.040_ | _0.041_ | _0.038_ | _0.042_ | _0.041_ | _0.041_ | _0.040_ | _0.040_ |
| _VIT_201s0127g00330-VviINO_ | _0.460_ | _0.482_ |  | _0.041_ | _0.041_ | _0.040_ | _0.040_ | _0.042_ | _0.038_ | _0.040_ | _0.040_ | _0.041_ | _0.041_ |
| _VIT_215s0048g00550-VviYAB7_ | _0.360_ | _0.381_ | _0.468_ |  | _0.031_ | _0.040_ | _0.041_ | _0.041_ | _0.040_ | _0.040_ | _0.041_ | _0.034_ | _0.035_ |
| _VIT_202s0154g00070-VviYAB6_ | _0.345_ | _0.396_ | _0.453_ | _0.151_ |  | _0.041_ | _0.041_ | _0.040_ | _0.041_ | _0.040_ | _0.042_ | _0.037_ | _0.037_ |
| _VIT_211s0016g05590-VviYAB5_ | _0.324_ | _0.345_ | _0.475_ | _0.367_ | _0.396_ |  | _0.040_ | _0.040_ | _0.041_ | _0.039_ | _0.030_ | _0.040_ | _0.041_ |
| _VIT_201s0011g00140-VviCRC_ | _0.475_ | _0.511_ | _0.547_ | _0.475_ | _0.475_ | _0.496_ |  | _0.041_ | _0.040_ | _0.034_ | _0.040_ | _0.041_ | _0.041_ |
| _AT1G08465-AthYAB2_ | _0.259_ | _0.317_ | _0.460_ | _0.374_ | _0.367_ | _0.374_ | _0.496_ |  | _0.041_ | _0.043_ | _0.039_ | _0.040_ | _0.040_ |
| _AT1G23420-AthINO_ | _0.482_ | _0.525_ | _0.288_ | _0.468_ | _0.489_ | _0.511_ | _0.568_ | _0.504_ |  | _0.039_ | _0.041_ | _0.041_ | _0.041_ |
| _AT1G69180-AthCRC_ | _0.532_ | _0.554_ | _0.597_ | _0.540_ | _0.525_ | _0.554_ | _0.245_ | _0.518_ | _0.626_ |  | _0.040_ | _0.040_ | _0.041_ |
| _AT2G26580-AthYAB5_ | _0.345_ | _0.374_ | _0.482_ | _0.381_ | _0.417_ | _0.158_ | _0.475_ | _0.374_ | _0.518_ | _0.532_ |  | _0.039_ | _0.041_ |
| _AT2G45190-AthYAB1(AFO_FIL)_ | _0.374_ | _0.410_ | _0.468_ | _0.230_ | _0.281_ | _0.403_ | _0.482_ | _0.374_ | _0.482_ | _0.540_ | _0.396_ |  | _0.037_ |
| _AT4G00180-AthYAB3_ | _0.353_ | _0.388_ | _0.475_ | _0.209_ | _0.266_ | _0.381_ | _0.496_ | _0.360_ | _0.489_ | _0.554_ | _0.396_ | _0.273_ |  |

Standard error estimates are given above the diagonal. The analysis involved 13 sequences. All positions containing gaps and missing data were eliminated. Evolutionary analyses were conducted in MEGA7.

**Table S4. INO orthologous proteins used for phylogenetic tree construction.**

| **Tree code** | **Accession number** | **Protein length (aa)** | **Organism** | **Organism Abbreviation** |
| --- | --- | --- | --- | --- |
| **AtrINO** | XM_006840616 | 186 | *Amborella trichopoda* | Atr |
| **AthINO** | [AT1G23420 (www.Arabidopsis.org)](http://www.ncbi.nlm.nih.gov/protein/18395240?report=genbank&log$=prottop&blast_rank=2&RID=8T73UFYA01N) | 231 | *Arabidopsis thaliana* | Ath |
| **RcoINO** | EEF37031.1 | 244 | *Ricinus communis* | Rco |
| **IniINO** | AAT42250.1 | 205 | *Impatiens niamniamensis* | Ini |
| **IsoINO** | [AAT42246.1](http://www.ncbi.nlm.nih.gov/protein/55739898?report=genbank&log$=prottop&blast_rank=2&RID=8T6RPCX101N) | 126 | *Impatiens sodenii* | Iso |
| **IwaINO** | [AAT42245.1](http://www.ncbi.nlm.nih.gov/protein/55584188?report=genbank&log$=prottop&blast_rank=4&RID=8T6RPCX101N) | 117 | *Impatiens walleriana* | Iwa |
| **AmaINO** | [AAS10181.1](http://www.ncbi.nlm.nih.gov/protein/41745691?report=genbank&log$=prottop&blast_rank=1&RID=8T77PNSC01N) | 235 | *Antirrhinum majus* | Ama |
| **AchINO** | [ADY39185.1](http://www.ncbi.nlm.nih.gov/protein/324455779?report=genbank&log$=prottop&blast_rank=1&RID=8T7HZDH701N) | 183 | *Annona cherimola* | Ach |
| **AsqINO** | [ADY39186.1](http://www.ncbi.nlm.nih.gov/protein/324455781?report=genbank&log$=prottop&blast_rank=2&RID=8T7HZDH701N) | 181 | *Annona squamosa* | Asq |
| **IgrINO** | [AAT42248.1](http://www.ncbi.nlm.nih.gov/protein/55584190?report=genbank&log$=prottop&blast_rank=1&RID=8T7W4F9001N) | 113 | *Impatiens grandis* | Igr |
| **IbaINO** | [AAT42247.1](http://www.ncbi.nlm.nih.gov/protein/55584189?report=genbank&log$=prottop&blast_rank=1&RID=8T80UBYB01N) | 72 | *Impatiens balsamina* | Iba |
| **SlyINO** | XP_010321151.2 | 192 | *Solanum lycopersicum* | Sly |
| **VviINO** | VIT_201s0127g00330.1 (www.cribi.unipd.it) | 176 | *Vitis vinifera* | Vvi |
| **OsaYABBY7** | BAF45808.1 | 169 | *Oryza sativa* | Osa |
| **CanINO** | AWQ60032.1 | 215 | *Capsicum annuum* | Can |
| **ParINO** | AKD43982.1 | 218 | *Prunus armeniaca* | Par |
| **PpeINO** | XP_007226582.1 | 221 | *Prunus persica* | Ppe |
| **PinINO** | AKD43983.1 | 221 | *Prunus incisa* | Pin |
| **GmaYABBY10** | XP_025985248.1 | 218 | *Glycine max* | Gma |
| **NalINO** | BAC82106.1 | 202 | *Nymphaea alba* | Nal |
| **NcoINO** | [BAC82107.1](https://www.ncbi.nlm.nih.gov/protein/34013380) | 201 | *Nymphaea colorata* | Nco |
| **CcaINO** | BAJ83621.1 | 209 | *Cabomba caroliniana* | Cca |
| **ZmaYAB4** | AIB05937.1 | 176 | *Zea mais* | Zma |
| **PvuINO** | ESW25742.1 | 237 | *Phaseolus vulgaris* | Pvu |
| **MtrINO** | AES96167.1 | 217 | *Medicago truncatula* | Mtr |
| **BraINOa** | XP_009115528.1 | 233 | *Brassica rapa* | Bra |
| **BsiINO** | QFZ79352.1 | 202 | *Bienertia sinuspersici* | Bsi |
| **CquINO** | XP_021742940.1 | 195 | *Chenopodium quinoa* | Cqu |
| **NbeINO1** | Niben101Scf09599g00012.1 (Solgenomics.net annotation as refined in Skinner et al., 2016) | 189 | *Nicotiana benthamiana* | Nbe |
| **NbeINO2** | Niben101Scf04287g04009.1 (Solgenomics.net annotation as refined in Skinner et al., 2016) | 189 | *Nicotiana benthamiana* | Nbe |

For each protein used for Fig. 2, accession number (genebank) and protein length in amino acids are given as well as information about the organism in which they were identified and the three letter species abbreviation. Alternatively databases from which sequence was derived are indicated beside the ID number.

**Table S5. List of primers used in this study**

| **Gene name** | **Primer sequence (5’-3’)** | **Annealing temp (°C)** | **Amplicon size (bp)** |
| --- | --- | --- | --- |
| *VviYAB6* | For: CACCGCAAATGACTTTTCTG | 56 | 178 |
|  | Rev: GGCTTCTCTGTGAGTTATATC |  |  |
| *VviYAB7* | For: TCATGCCGGTGCGAGGAGTC | 64 | 158 |
|  | Rev: GGCCTCTCTGTGACTTATGC |  |  |
| *VviYAB2a* | For: GATATTACCCACAGGGAAGC | 60 | 121 |
|  | Rev: CTGCAAATGCCTGATCCAAC |  |  |
| *VviYAB2b* | For: GATCTTCAGAAGGAGAACCT | 58 | 152 |
|  | Rev: ACCCTTTGCTTTTTCTCTTG |  |  |
| *VviYAB5* | For: GTTCAGGCACCCAACTACAC | 58 | 127 |
|  | Rev: GGCGATTCACAATCCTTTCC |  |  |
| *VviCRC* | For: AAGTAGCCAATCCTGAGATA | 55 | 118 |
|  | Rev: CATTCATTGTTACTGCTCTC |  |  |
| *actin2 grape* | For: GCACCCTTCGCACGATATGA | 58 | 117 |
|  | Rev: TGACGCAAGGCAAGGACTGA |  |  |
| *VviINO.1* | For: CAGCGAGCTCCATCAGCTTAT | 60 | 63 |
|  | Rev: TCAGCCTTAAGCCTTCTGATCTCT |  |  |
|  | Probe: ACCGCTTCATCAAAG |  |  |
| *VviINO.2* | For: AACCGCTTCATCAAGTAACTAACAAA | 60 | 67 |
|  | Rev: AGCCTTCTGATCTCTTCTCTGCTATT |  |  |
|  | Probe: TTGGTTATATGCCAACTAGT |  |  |
| *VviActin2* | Assay ID: Vv04085614_g1 (TaqMan assay from https://www.thermofisher.com/taqman-gene-expression/product/Vv04085614_g1?CID=&ICID=&subtype=) | 60 | 98 |
| *AthActin2* | Assay ID: At02335270_gH (TaqMan assay from https://www.thermofisher.com/taqman-gene-expression/product/At02335270_gH?CID=&ICID=&subtype=) | 60 | 148 |
| *VviINO-1 forward* | For: ATGTCAACACTGAACCATCTCT | 66 | 531/612 |
| *VviINO-4 reverse* | Rev: CTACCTTTCCTGGCTACAG |  |  |
| *BamVviINOF* | For: GCAGGGATCCTCCTGAAGAACATGTCAACACTGAACCATCTCT | 62 | 562/643 |
| *XbaVviINOR* | Rev: CCAGTCTAGACTACCTTTCCTGGCTACAG |  |  |
| *4CKfor* | For: GAGTCAATGCATGTCTCTTC | 56 | 865/946 |
| *4CKrev* | Rev: GCTTACAGCTCATAGAGAC |  |  |
| *ino1-genfor* | For: CTCACAAGGAAGCTTTCAGC | 55 | 419 |
| *ino1-genrev* | Rev: GCGATGCTATTTGGTCTAGT |  |  |
